# 分离技术在新型冠状病毒研究和防疫检测中的应用

**DOI:** 10.3724/SP.J.1123.2021.03022

**Published:** 2021-07-08

**Authors:** Linsen LI, Chao ZHU, Xinying ZHAO, Feng QU

**Affiliations:** 1.北京理工大学生命学院, “分子医学与生物诊疗”工业和信息化部重点实验室, 北京 100081; 1. School of Life Science, Beijing Institute of Technology, “Molecular Medicine and Biological Diagnosis” Key Laboratory of Ministry of Industry and Information Technology, Beijing 100081, China; 2.北京电子科技职业学院, 北京 100176; 2. Beijing Polytechnic, Beijing 100176, China

**Keywords:** 分离技术, 亲和色谱, 尺寸排阻色谱, 液相色谱, 磁珠分离, 离心, 微纳分离, 电泳, 新型冠状病毒, separation technology, affinity chromatography (AC), size exclusion chromatography (SEC), liquid chromatography (LC), magnetic bead separation, centrifugation, micro/nano-separation, electrophoresis, severe acute respiratory syndrome coronavirus 2 (SARS-CoV-2)

## Abstract

新型冠状病毒肺炎(COVID-19)疫情的爆发给世界公共卫生安全带来前所未有的挑战。随着新型冠状病毒(SARS-CoV-2)相关研究的不断深入,众多分析检测技术相继被应用,推动了病毒检测、疫苗和创新疗法的研发,从而使疫情早日得到控制。分离技术作为生命科学、医学、药学领域的关键技术,操作简单,分离效率高,选择性强,在新型冠状病毒的分离、检测、诊疗及防疫中起到不可替代的作用。该文以SARS-CoV-2或COVID-19为关键词在ISI Web of Science中进行主题检索,归纳了2020年度新型冠状病毒相关的研究论文,简要介绍主要的研究方向,并对国际顶级学术期刊*Nature*, *Science*, *Cell*的论文发表情况进行了统计。通过检索影响因子较高的期刊,综述了新型冠状病毒研究中主要应用的分离技术,并从亲和色谱和尺寸排阻色谱、液相色谱、磁珠分离、离心、微纳分离以及电泳6个方面进行说明。综述统计了亲和色谱和尺寸排阻色谱纯化的病毒相关蛋白,并介绍了其在新型冠状病毒传播、感染机制以及药物筛选中的应用;介绍了液相色谱对病毒候选药物评估以及复杂基质中单一成分的鉴定;介绍了磁珠分离在细胞分离、核酸提取和免疫学检测中的应用;介绍了离心对病毒颗粒、细胞以及血清的分离;介绍了微纳分离结合其他技术以实现病毒蛋白的高灵敏检测;简要介绍了电泳在聚合酶链式反应(PCR)产物分析中的应用。该文综述了2020年度新型冠状病毒研究和防疫检测中分离技术的应用情况,分析了分离技术在新型冠状病毒检测中发挥的作用,旨在为从事分离研究的科研工作者提供一些参考。

## Abstract (en)

The novel coronavirus disease 2019 (COVID-19) outbreak has brought to light unprecedented challenges to global public health security. Researchers have devoted their efforts to in-depth research on severe acute respiratory syndrome coronavirus 2 (SARS-CoV-2) to bring the epidemic under control as rapidly as possible. Among the many areas of burgeoning SARS-CoV-2 related research, various analytical technologies have been applied to the advancement of virus detection, and development of vaccines and innovative therapies. Separation technologies with the merits of simple operation, high separation efficiency, and high selectivity, have become widely used and are key to progress in life science, medicine, pharmaceutical discovery and development, and other fields. Separation technologies have played an irreplaceable role in the isolation, detection, diagnosis, treatment, and prevention of this novel coronavirus. In this review, an overview of the relevant literature is presented from ISI Web of Science spanning Jan. 1st, 2020-Dec. 31, 2020, using “SARS-CoV-2” or “COVID-19” as keywords. The top 20 research directions are summarized, based on papers published in high impact international journals (e. g. *Nature*, *Science*, and *Cell*). Incorporating the impact of published papers, this review summarizes the primary separation technologies applied in these coronavirus studies, and discusses contributions of the following six technologies: affinity chromatography and size exclusion chromatography, liquid chromatography, magnetic bead separation technology, centrifugal technology, micro/nano-separation technology, and electrophoresis. First, affinity chromatography and size exclusion chromatography are discussed, which are the most frequently used protein purification techniques in *Nature*, *Science*, and *Cell*. The SARS-CoV-2 related proteins purified by affinity chromatography and size exclusion chromatography are summarized, and their applications in coronavirus transmission, infection mechanisms, and drug screening are introduced. Subsequently, high performance liquid chromatography (HPLC) is introduced, which is mainly employed for assessing the purity of candidate drugs. Liquid chromatography-tandem mass spectrometry (LC-MS/MS) incorporates the strengths of HPLC and MS, offering both high separation efficiency and structural analysis capabilities with extended applications. LC-MS/MS has been applied to characterization of the binding of SARS-CoV-2 related proteins to potential inhibitors, and to metabolic analyses of candidate drugs. In SARS-CoV-2 nucleic acid tests, magnetic bead separation technology plays a crucial role in the separation of novel coronaviruses. In combination with other analytical techniques, magnetic bead separation technology can be applied to cytological analyses and immunological detection by functionalization of bead surfaces. Centrifugal technology is undoubtedly the most basic separation technology. It has been employed in almost all SARS-CoV-2 related researches. By controlling centrifugation speed, centrifugal technology can rapidly isolate virus particles or cultured cells from complex samples. Micro-nano separation technologies, such as microfluidics, offer advantages including small size, low sample consumption, rapid diffusion, and large surface area. In general, microfluidic technologies are often used in combination with other technologies to realize highly sensitive detection of SARS-CoV-2 related proteins. Finally, the applications of electrophoresis are introduced, which commonly engages in the analysis of polymerase chain reaction (PCR) products. In novel coronavirus studies, the application of electrophoresis has been relatively limited but has potential with further development to contribute significantly to future research. In conclusion, this review summarizes the contributions of six primary separation technologies to novel coronavirus studies, including epidemic detection and prevention, analyzes the main problems facing coronavirus detection efforts, and discusses the role of separation technologies in addressing these problems, with the aim of providing references for broader application of separation technologies.

2020年1月爆发的新型冠状病毒肺炎(COVID-19)在短时间内迅速蔓延全球,给世界公共卫生体系带来了前所未有的挑战^[1]^。国际病毒分类委员会将引发新冠肺炎的病毒命名为严重性呼吸综合征冠状病毒2(SARS-CoV-2),它是一种单股正链RNA病毒(*β*-冠状病毒属),早期通过表面凸起的刺突糖蛋白(S蛋白)结合受体细胞的血管紧张素转换酶2(ACE2),后经细胞内2型跨膜丝氨酸蛋白酶裂解ACE2并激活S蛋白来促进病毒吸收,进而引起病变^[2]^。目前,疫情已持续1年多,全球累计确诊病例超1.18亿例,且仍呈增加趋势。为了尽快控制疫情,全球科研和临床医护人员联手攻关,从病毒的传播途径、感染致病机制、快速检测诊断、疫苗开发等多方面开展研究,在短时间内取得了一系列突破进展。分离技术作为生命科学、医学、药学等领域的关键技术,在新型冠状病毒研究中发挥了不可替代的作用。其中,以色谱为代表的现代分离技术,在实现病毒微量蛋白质纯化的同时能保持蛋白质活性和分子结构完整,适用于发病机制、疫苗研发、临床治疗等研究。而以离心为代表的传统分离技术,是新型冠状病毒研究的基础技术手段,方法简单快捷,可初步分离病毒、细菌等大颗粒物质。

本文以“COVID-19”或“SARA-CoV-2”为关键词,在ISI Web of Science数据库中进行主题检索,截至2020年12月31日为止,相关期刊论文已达26160篇,研究方向包括普通内科学、公共环境卫生、传染病学、药理学、分子生物学、化学等(见图1)。其中,国际顶级学术期刊*Nature*, *Science*和*Cell*分别发表新型冠状病毒相关论文62、58和43篇,论文中应用的分离技术主要有亲和色谱(AC)和尺寸排阻色谱(SEC)、液相色谱、磁珠分离和离心,研究内容涉及新型冠状病毒的传播、溯源、治疗药物、疫苗研发、临床防治和疫情防控等方面。分析化学类专业期刊发表相关论文有限,代表性期刊*Analytical Chemistry*在2020年全年仅发表14篇新型冠状病毒研究论文,其中2篇报道采用微纳分离技术对病毒进行高灵敏检测。

**图 1:**
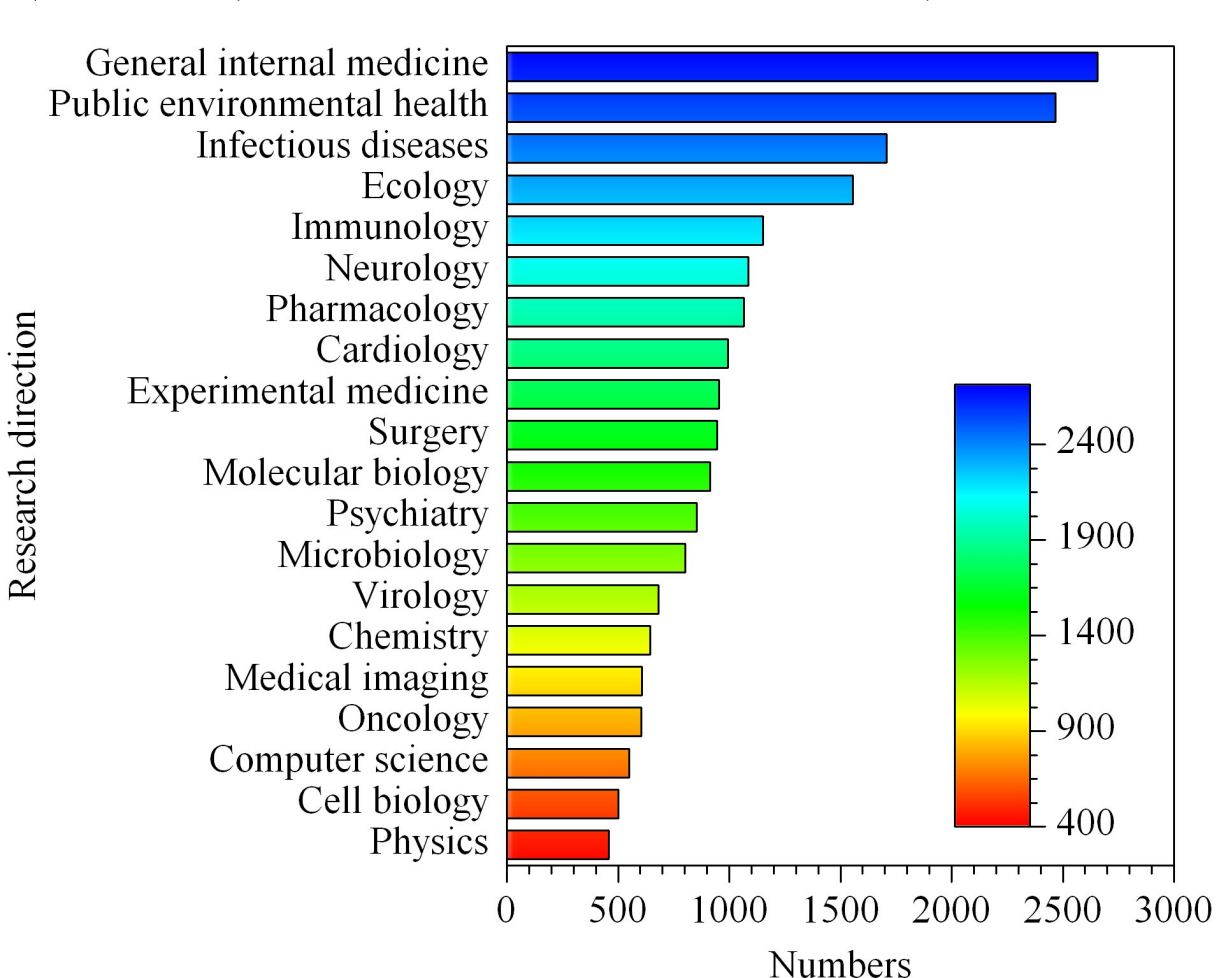
2020年新型冠状病毒研究文献数量分布

本文总结了分离技术在新型冠状病毒研究、检测和防疫中的应用,从亲和色谱和尺寸排阻色谱、液相色谱、磁珠分离、离心、微纳分离和电泳6个方面进行介绍。

## 1 AC和SEC

亲和色谱和尺寸排阻色谱是*Nature*, *Science*和*Cell*期刊报道文献中使用最频繁的蛋白质纯化技术。其中,AC是通过生物分子与功能配基的特异性作用对目标蛋白质进行分离纯化,是实验室中生物大分子分离纯化最具特异性和高效性的技术,但配基昂贵且偶联技术复杂,限制了其应用;SEC是根据相对分子质量差异对待测物中各组分进行分离,优势在于分辨率高,操作简单,但对于样品纯度要求很高,因此常用于亲和色谱后蛋白质的进一步纯化。在当前新型冠状病毒研究中,AC与SEC常被用于纯化病毒表面S蛋白、ACE2、S蛋白受体结合域(RBD)等。

### 1.1 新型冠状病毒传播研究

Shang等^[3]^使用镍离子金属螯合亲和色谱(Ni-NTA)对多聚组氨酸标签(His_6_-tag)标记的SARS-CoV-2和SARS-CoV的RBD以及ACE2胞外域分离纯化,使用Protein A亲和色谱纯化重组人APOM蛋白(Fc-tag)标记的ACE2,随后用SEC对蛋白质进一步纯化。通过比较分析ACE2对SARS-CoV-2、SARS-CoV以及蝙蝠冠状病毒RaTG13的识别差异,揭示SARS-CoV-2在动物和人类之间的潜在传播。以同样方法分离纯化SARS-CoV-2、SARS-CoV和中东呼吸综合征冠状病毒(MERS-CoV)的RBD以及ACE2胞外域后,Shang等^[4]^对比分析ACE2与3种病毒RBD的结合差异,为病毒干扰策略研究提供参考。

### 1.2 病毒感染机制研究

Yan等^[5]^通过链霉亲和素突变体(Strep-Tactin)琼脂糖凝胶和SEC纯化了亲水性多肽标签(FLAG-tag)标记中性氨基酸转运蛋白(B^0^AT1)和链霉亲和素结合肽标签(Strep-Tag)标记ACE2,研究在有无RBD的情况下,ACE2与B^0^AT1复合物的晶体结构差异,推动病毒感染的分子基础研究。Hillen等^[6]^利用组氨酸标记(HisTrap)亲和色谱柱纯化冠状病毒复制和转录的主要RNA聚合酶(Nsp 12)及其辅助因子Nsp 7和Nsp 8,分析这些蛋白质与RNA依赖性聚合酶的结构,应用于病毒RNA复制研究。

### 1.3 新型冠状病毒的药物筛选及疫苗设计

Gao等^[7]^用Ni-NTA亲和色谱柱纯化了Nsp 12、Nsp 7和Nsp 8,解析了该聚合酶与抗病毒药物瑞德西韦(remdesivir)的结合方式。Riva等^[8]^利用Ni-NTA亲和色谱柱对SARS-CoV-2的木瓜蛋白酶样蛋白酶(PLpro)和主蛋白酶(Mpro)进行纯化,从约12000个临床阶段的小分子药物中筛选出13种具有治疗效果的小分子抑制剂。Yuan研究组^[9]^将Protein G亲和柱用于患者体内SARS-CoV特异性人类单克隆抗体(CR3022)的纯化,研究发现CR3022可有效结合SARS-CoV-2的RBD蛋白,是一种潜在的抗病毒药物。Ju等^[10]^使用Protein A亲和柱对SARS-CoV-2感染者B细胞中RBD特异性单克隆抗体进行纯化,从206种纯化的单克隆抗体中发现抗SARS-CoV-2中和活性抗体,且该抗体基本不结合SARS-CoV和MERS-CoV。

表1统计了上述AC和SEC纯化的多种新型冠状病毒相关蛋白质,并列举了纯化使用的蛋白标签。

**表 1:** 亲和色谱和尺寸排阻色谱在新型冠状病毒相关蛋白纯化中的应用

Target protein	Tag	Purification	Ref.
SARS-CoV-2 RBD (residues 319-535), SARS-CoV RBD (residues 306-521), MERS-CoV RBD (residues 367-588)	C-terminal (His)_6_-tag	Ni-NTA, SEC	[4]
SARS-CoV-2 RBD (residues 319-541)	C-terminal (His)_6_-tag	Ni-NTA, SEC	[9]
SARS-CoV RBD (residues 306-527)			
SARS-CoV-2 RBD (residues 331-524)	C-terminal (His)_8_-tag, AviTag	Ni-NTA, SEC	[11]
RBD (residues 319-541)	C-terminal (His)_6_-tag	Ni-NTA, SEC	[12]
Spike protein	C-terminal (His)_8_-tag	Ni-NTA, SEC	[13,14]
Spike protein	C-terminal (His)_6_-tag	Ni-NTA, SEC	[9]
S-ECD	C-terminal FLAG-tag	Anti-FLAG M2 affinity resin, SEC	[15]
SARS-CoV-2 S 2P ectodomain	C-terminal (His)_8_-tag, AviTag	Ni-NTA, SEC	[11]
ACE2 peptidase domain (residues 1-615)	C-terminal (His)_6_-tag	Ni-NTA, SEC	[3,4]
	C-terminal Fc-tag	Protein A column, SEC	
ACE2	strep tag	Strep-Tactin Sepharose, SEC	[5]
IgGs	no tag	Protein G column, SEC	[9]
CR3302 Fab	no tag	CaptureSelect^TM^ CH1- 50 XL Pre-packed Column, SEC	
IgG1	no tag	Protein A column	[10]
BD23-Fab	no tag	Protein A column (remove Fc region), SEC	[14]
PLpro	N-terminal (His)_6_-tag	Ni-NTA, SEC	[8]
Mpro	N-terminal (His)_6_-tag	Anion-exchange, SEC	
B^0^AT1	N-terminal FLAG-tag	Strep-Tactin Sepharose, SEC	[5]
Nsp 12, Nsp 7, Nsp 8	N-terminus (His)_6_-tag	HisTrap column	[6]
Nsp 12	C-terminus (His)_10_-tag	Ni-NTA, Hitrap Q ion-exchange column, SEC	[7]
Nsp 7, Nsp 8	N-terminus (His)_6_-tag		
Nsp 12	C-terminus (His)_10_-tag	Ni-NTA, SEC	[16]
Nsp 7, Nsp 8	N-terminus (His)_6_-tag		

RBD: receptor binding domain; S-ECD: S protein extracellular domain; IgGs: immunoglobulin G; Fab: antigen-binding fragment; PLpro: papain-like protease; Mpro: main protease; B^0^AT1: neutral amino acid transporter; Nsp: non-structural protein; Ni-NTA: nickel nitrilotriacetic acid.

## 2 液相色谱

高效液相色谱(HPLC)具有高效、快速、灵敏度高、重复性好等特点,是化合物纯度鉴定和生物大分子分析的通用分析方法。液相色谱-串联质谱(LC-MS/MS)在保留色谱技术高效分离优势的同时,通过MS获得待测组分丰富的结构信息,在药物代谢、复杂基质单一成分鉴定、代谢组学研究等方面凸显优势^[17]^。

### 2.1 新型冠状病毒候选药物评估

Zhang等^[18]^报告了冠状病毒药物靶标Mpro与*α*-酮酰胺抑制剂复合物的X射线结构,通过LC-MS/MS分析确定*α*-酮酰胺抑制剂13a和13b具有明显的肺向性。Jin等^[19]^利用计算机辅助药物设计筛选Mpro抑制剂,测定了10000多种药物或活性化合物,并利用LC-MS/MS分析确定依布硒啉、PX-12和卡莫呋对Mpro的共价结合。Ma等^[20]^将非变性质谱(native MS)用于Mpro与4种潜在抑制剂(波普瑞韦、GC-376以及钙蛋白酶抑制剂II和XII)的结合表征,结果发现此4种化合物均能明显抑制SARS-CoV-2在细胞内复制。Maisonnasse等^[21]^基于LC-MS/MS研究了羟氯喹(HCQ)在体外和SARS-CoV-2感染猕猴中的抗病毒活性,通过定量分析血清、血液以及肺组织中HCQ含量,发现HCQ没有明显的预防感染能力。

### 2.2 复杂基质单一成分鉴定

Dai研究组^[22]^针对SARS-CoV-2主要蛋白酶Mpro设计合成了两种先导化合物11a和11b,通过HPLC鉴定其纯度,分别为99.88%和99.20%,这两种化合物在体内均表现出有效的抗SARS-CoV-2感染活性,可作为候选药物用于后续临床研究。Monteil等^[23]^使用HPLC鉴定了鼠源重组ACE2,并比较了鼠源重组与人重组ACE2在SARS-CoV-2抗感染方面的差异。结果表明,人重组ACE2可显著降低细胞和多种人体器官模型的病毒感染,而鼠源重组ACE2则没有此效果。在对患病程度不同的新冠病人及健康人员血清进行蛋白质组学和代谢组学分析后,Shen等^[24]^利用超高效液相色谱-串联质谱(UPLC-MS/MS)从血清中分离鉴定并定量了894种蛋白质和941种代谢物(包括36种药物及其代谢物),揭示了重症患者血清中特征蛋白质和代谢产物的变化,为疾病严重程度评估提供参考。

## 3 磁珠分离

磁珠分离技术可通过磁性颗粒表面修饰的官能团或特异性抗体,从复杂基质中选择性结合靶标分子,并在外磁场辅助下实现目标物富集,其分离效率高、重复性好,但提取效率低且价格较高。当前,磁珠分离技术仍是新型冠状病毒分离的主要方法,且有多款基于磁微粒-化学发光的检测试剂盒通过国家药监局审批。该试剂盒采用双抗原夹心原理,形成化学发光剂/抗原-抗体-磁颗粒/抗原复合物,在磁场辅助下分离结合状态和游离状态的化学发光剂标记物,随后加入发光促进剂进行发光反应,通过对发光强度的检测进行定量或定性分析。磁珠分离技术还能与病毒核酸自动化提取设备配套使用,对大批量样本的核酸进行提取,方法操作简单,极大节约了时间和人工成本,可显著提高核酸的检测效率^[25]^。

在新型冠状病毒研究中,磁珠分离技术主要应用于细胞分离、核酸提取和免疫学检测。Cao等^[14]^利用免疫磁珠从外周血单核细胞(PBMC)中负选分离到B细胞,并用结合肿瘤坏死因子受体超家族7(CD27)抗体的磁珠进一步分离得到CD27^+^记忆B细胞,通过高通量单B细胞测序对新冠康复期患者体内中和抗体进行鉴定。Zhao等^[26]^在磁性纳米颗粒上涂覆了一层聚(氨基酯)-羧基,利用核酸与羧基之间强相互作用提取SARS-CoV-2的RNA。该方法将病毒遗传物质裂解、提取和结合步骤组合到一起,得到的纳米颗粒-RNA复合物不需要额外洗脱即可引入后续的反转录聚合酶链式反应(RT-PCR),极大提高COVID-19的诊断效率。Fabiani研究组^[27]^以磁珠为免疫载体,碱性磷酸酶为免疫标记二抗,开发了一种唾液中SARS-CoV-2快速检测的电化学免疫分析法。所建方法可用于唾液临床样本中S蛋白和核衣壳蛋白(N蛋白)的检测,检出限分别为19 ng/mL和8 ng/mL。

## 4 离心

离心技术操作简易,成本较低,是通过控制离心机转速实现样品中蛋白质、核酸及细胞亚组分的分离。作为最基础的分离技术,离心技术在新型冠状病毒检测中可用来分离血浆中的血清,用于抗体和抗原的检测。在新型冠状病毒实验室研究环节,通过离心技术可分离鼻咽拭子中颗粒物质以及未消化的死细胞和纤维碎片,并反复离心清洗来沉淀纯化病毒核酸。相比于普通离心技术,超速离心技术具有更强的离心力场,可迅速实现小颗粒(如病毒颗粒、蛋白质等)的沉降分离。Bao等^[28]^使用超速离心从Vero E6病变细胞中沉淀获得病毒颗粒,研究了SARS-CoV-2在表达人ACE2转基因小鼠中的致病性。Zost等^[29]^将超速离心用于S蛋白假型慢病毒的分离,来测定两种单克隆抗体的中和能力,结果显示假病毒比野生型病毒具有更敏感的中和表型,证明在单克隆抗体效果测定中使用活病毒的必要性。此外,聚蔗糖-泛影葡胺(Ficoll-hypaque)密度梯度离心技术也被用于分离PBMC,以研究普通冠状病毒和SARS-CoV-2的免疫学差异^[30]^。

## 5 微纳分离

材料科学和电子技术的进步带动了微纳分离技术发展,使其成为生物样品分析的重要技术手段。微流控技术具有微型尺寸、样品量小、快速扩散、比表面积大等优势,常与其他技术结合,用于SARS-CoV-2相关蛋白质的高灵敏检测。Lin等^[31]^开发了一种微流控免疫芯片,结合自制荧光检测器分析检测了SARS-CoV-2 3种生物标志物(IgG、IgM和抗原)。该方法快速、灵敏、便捷,在15 min内即可完成SARS-CoV-2检测。Xiong研究组^[32]^基于环介导等温扩增技术(LAMP),开发了一种便携式LAMP-微流控集成系统,该系统使用小型圆盘状微流控芯片(81 mm),可在40 min内同时识别7种已知人类冠状病毒。Funari等^[33]^将电沉积方法用于一种光微流体传感平台开发,通过比较金纳米钉在抗原-抗体结合时局部折射率变化,在30 min内实现1 μL血清样品中SARS-CoV-2 S蛋白特异性抗体测定,检出限低至0.08 μg/L(~ 0.5 pmol/L)。Tan研究组^[34]^提出了一种便携式化学发光微流体的酶联免疫吸附测定(ELISA)技术,从候选蛋白质中筛选出对SARS-CoV-2 S1蛋白具有高亲和力和特异性的D006作为校准抗体,在15 min内可对血清中低至2 ng/mL的S1特异性IgG进行定量评估。此技术还被用于S1和N蛋白的高灵敏检测(40 min),检出限分别为4 pg/mL和62 pg/mL。Lakshmanan等^[35]^将微流控芯片技术用于体外抗凝治疗的止血效果评估,设计了一种具有两条正交通道的微流控装置,其中一条通道作为血管模型,另一条通道用来模拟损伤部位止血塞的形成,用于识别潜在的抗凝靶点和试验药物,以此减轻COVID-19相关的血栓并发症。

## 6 电泳

电泳技术利用带电粒子在电场中移动速率不同而达到分离的技术,是生命分析领域重要分离手段之一。然而,在新型冠状病毒研究中电泳的许多应用均可被分子生物学方法替代,且在实际检测中相比于自动化设备和商品化试剂盒,其技术要求高,因而应用有限,亟待后续研究拓展^[36]^。其中,琼脂糖凝胶电泳(AGE)是应用较多的电泳技术,常被用于聚合酶链式反应(PCR)产物分析。Kim等^[37]^对SARS-CoV-2的RNA基因组结构进行了解析,并用AGE方法验证了测序发现的亚基因组RNA(sgRNAs),进而揭示基因在RNA基因组上的确切位置。Meza-Robles等^[38]^提出一步嵌套式RT-PCR,不需要实时热循环仪,通过AGE即可实现新型冠状病毒的定量检测。该技术使用4个物种特异性的诊断引物,可同时实现特定区域扩增和阳性对照。Si等^[39]^对2188例疑似COVID-19患者的鼻咽拭子样本进行分析,使用RT-PCR检测SARS-CoV-2, PCR片段分析结合毛细管电泳检测12种病毒,以研究SARS-CoV-2和常见呼吸道病毒的流行病学规律,以此提高疑似COVID-19患者的诊断效率。

## 7 结论

新型冠状病毒研究已取得一定进展,分离技术在其中发挥着重要作用。其中,AC和SEC广泛用于病毒相关蛋白质纯化中;精密仪器制造业的发展,促使HPLC分离效率的提升,是完成高灵敏度、快速解决病毒代谢组学分析的关键手段;新材料和电子技术的进步,带动磁珠和微纳分离技术的灵敏度提升和应用范围拓展,尤其是对化学试剂消耗降低,实现了对环境的友好,是解决细胞分析、核酸分离和免疫学检测的首选技术;传统的离心技术在病毒颗粒和细胞分离中发挥着不可替代的作用;电泳技术主要用于PCR产物分析。

当前,新型冠状病毒检测的难点主要集中在“假阳性”和“假阴性”结果上。作为新型冠状病毒检测的“金标准”,核酸检测能检测出处于窗口期的患者,但病毒载量差异、样品采集、诊断试剂质量、实验操作等问题会导致“假阴性”结果;抗体检测操作简单快捷,可作为核酸检测假阴性的有效补充,但其对灵敏度和特异性要求高,灵敏度低会产生“假阴性”结果,特异性低则会产生“假阳性”结果。分离技术作为新型冠状病毒检测的关键技术,可分离纯化复杂基质中病毒颗粒、核酸和蛋白质,减少基质中干扰物质带来的“假阳性”结果;同时,分离技术与新型检测技术结合,可进一步提高病毒相关蛋白质检测的灵敏性和特异性,确保检测结果准确可靠。

综上所述,分离技术在新型冠状病毒研究和防疫检测中应用广泛。这些分离技术各具特色,根据实验需要选用合适的分离技术,灵活组合,能有效推动新型冠状病毒检测、疫苗和创新疗法的研发,使疫情早日得到控制。
